# Autism Spectrum Disorders and Schizophrenia: Meta-Analysis of the Neural Correlates of Social Cognition

**DOI:** 10.1371/journal.pone.0025322

**Published:** 2011-10-05

**Authors:** Gisela Sugranyes, Marinos Kyriakopoulos, Richard Corrigall, Eric Taylor, Sophia Frangou

**Affiliations:** 1 Department of Child and Adolescent Psychiatry, Institute of Psychiatry, King's College London, London, United Kingdom; 2 Section of Neurobiology of Psychosis, Department of Psychosis Studies, Institute of Psychiatry, King's College London, London, United Kingdom; 3 Department of Child and Adolescent Psychiatry and Psychology, Institute of Neuroscience, University of Barcelona, Barcelona, Spain; 4 Child and Adolescent Mental Health Services, South London and Maudsley NHS Foundation Trust, London, United Kingdom; University of Adelaide, Australia

## Abstract

**Context:**

Impaired social cognition is a cardinal feature of Autism Spectrum Disorders (ASD) and Schizophrenia (SZ). However, the functional neuroanatomy of social cognition in either disorder remains unclear due to variability in primary literature. Additionally, it is not known whether deficits in ASD and SZ arise from similar or disease-specific disruption of the social cognition network.

**Objective:**

To identify regions most robustly implicated in social cognition processing in SZ and ASD.

**Data Sources:**

Systematic review of English language articles using MEDLINE (1995–2010) and reference lists.

**Study Selection:**

Studies were required to use fMRI to compare ASD or SZ subjects to a matched healthy control group, provide coordinates in standard stereotactic space, and employ standardized facial emotion recognition (FER) or theory of mind (TOM) paradigms.

**Data Extraction:**

Activation foci from studies meeting inclusion criteria (n = 33) were subjected to a quantitative voxel-based meta-analysis using activation likelihood estimation, and encompassed 146 subjects with ASD, 336 SZ patients and 492 healthy controls.

**Results:**

Both SZ and ASD showed medial prefrontal hypoactivation, which was more pronounced in ASD, while ventrolateral prefrontal dysfunction was associated mostly with SZ. Amygdala hypoactivation was observed in SZ patients during FER and in ASD during more complex ToM tasks. Both disorders were associated with hypoactivation within the Superior Temporal Sulcus (STS) during ToM tasks, but activation in these regions was increased in ASD during affect processing. Disease-specific differences were noted in somatosensory engagement, which was increased in SZ and decreased in ASD. Reduced thalamic activation was uniquely seen in SZ.

**Conclusions:**

Reduced frontolimbic and STS engagement emerged as a shared feature of social cognition deficits in SZ and ASD. However, there were disease- and stimulus-specific differences. These findings may aid future studies on SZ and ASD and facilitate the formulation of new hypotheses regarding their pathophysiology.

## Introduction

Autism and psychotic disorders have historically been considered as related diagnostic entities. One of the innovations of the DSM-III in the late 1970s was to separate autism spectrum disorders (ASD) from schizophrenia (SZ) into different diagnostic categories. Although this distinction has many practical advantages, it is currently being reconsidered in view of emerging evidence about common neurobiological processes in both disorders [1]–[3].

Impairment in social cognition is a cardinal feature of the clinical presentation of both ASD and SZ [4]–[8]. The term social cognition refers to a complex set of processes subserving adaptive social interaction which depend on “theory of mind”, or in other words, the ability to make correct attributions of the mental states of others [9]–[10]. Theory of mind (ToM) broadly refers to three types of attributions: attribution of epistemic mental states (e.g. beliefs), attribution of intentions or motivations and attribution of affective states. A range of tasks have been developed to map on these core mentalising domains. Facial emotion recognition (FER) relates to the ability to infer the emotional state of others, and although it measures a core dimension of ToM, it is usually mentioned separately. We will follow this convention here for ease of reference to the existing literature. Similarly, we will use the term ToM to collectively refer to tasks of epistemic (e.g. false beliefs) or intention attribution and tasks that involve more than one ToM domain (e.g. the attribution of mental states and intentions to animated geometric figures).

Neurobiological models of social cognition implicate an extended neural network in processing social stimuli [11]–[15]. Regions most consistently involved are the medial and ventrolateral prefrontal cortex (PFC), ventral temporal regions around the superior temporal sulcus (STS), occipitotemporal regions (particularly within the fusiform gyrus), the temporoparietal junction (TPJ), and limbic structures, especially the amygdala. These regions are interconnected and have additional connectivity with somatosensory cortices and subcortical structures such as the thalamus. There is emerging consensus for relative regional specialization within this extended network. Involvement of the medial PFC in social cognition is elicited by multiple tasks that require conscious attribution or judgment of mental states, dispositional traits or intentions of one's self or of others [16]. Engagement of the ventrolateral PFC relates primarily to the contextual or social appropriateness of responses to social cues [17]–[19]. Regions surrounding the TPJ have systematically been associated with social cognition tasks requiring participants to “think about other people's thoughts” or in other words, to take a third person perspective about others' affective or cognitive states [20], [21]. Activation in regions around the STS has been reliably associated with salient biological movement, including changeable characteristics of human facial features, which can be used to infer affective or intentional states [22], [23]. Similarly, somatosensory cortices are thought to contribute to social cognition by invoking or “mirroring” internal representations of affective states [24]. The amygdala is thought to contribute to social cognition by mediating arousal [25] or biological salience [26] associated with stimuli.

Current reviews of the neural basis of social cognition deficits in SZ [27], [28] and ASD [29] have implicated most, if not all the regions within this network. However, the current literature has two significant limitations. Firstly, existing reviews, which are mostly narrative, highlight the variability in the findings from individual studies, but do not provide an integrated model of the functional neuroanatomy of social cognition in either SZ or ASD. Secondly, there are no neuroimaging studies to date directly comparing patients with ASD or SZ, with the single exception of Pinkham et al [30]. The authors compared SZ and ASD patients while performing a functional magnetic resonance imaging (fMRI) task requiring participants to judge the trustworthiness of human faces; both patient groups showed reduced activation in the amygdala and ventrolateral PFC.

The aims of this study were to synthesize existing fMRI data using a meta-analytic approach in order to (a) identify regions most robustly implicated in social cognition processing in SZ and ASD and (b) to draw inferences about differences and similarities in the neural correlates of social cognition between the two disorders. Our key predictions were that during tasks of social cognition both disorders would be associated with reduced engagement within PFC regions associated with mentalising, and that similar functional disruption would also be observed in limbic regions, particularly the amygdala. We also hypothesized that in SZ, PFC dysfunction would be associated with reduced down-regulation of more posterior brain regions involved in the attribution of salience to biological social cues (e.g. facial affect).

## Materials and Methods

### Data sources and inclusion criteria

The study design and report adhered to the PRISMA Statement guidelines (supporting information Table S1 and Figure S1). The search method and inclusion criteria were specified in advance, informed by existing meta-analyses [1], [31], [32]. All identified articles were reviewed for eligibility by at least two authors, and decisions for inclusion were made by consensus. Data was extracted independently from each study by the first author, and was subsequently reviewed by a second author.

Studies investigating FER or ToM in subjects with ASD (including autism, Asperger's Syndrome and pervasive developmental disorder not otherwise specified) or SZ were identified through a computerized literature search using Medline. We reviewed all papers in English language published up to 2010. The following search keywords were employed: “autism”, “schizophrenia”, “asperger”, and “facial emotion”, “emotional processing”, “social cognition”, “theory of mind”, “mentalization”, “irony”, “empathy”, “fMRI” and their combinations and differing terminations, as well as terms specifying individual facial affect (fear, happiness, sadness, anger and disgust). The reference lists of these papers were searched for additional articles.

In order to meet inclusion criteria, studies were required to (a) report comparisons between ASD or SZ patients and matched healthy controls, (b) employ functional magnetic resonance imaging (fMRI), (c) use image subtraction methodology to identify foci of task-related neural changes contrasting an active and control condition, and (d) report their results in standard stereotactic coordinates (either Talairach or Montreal Neurological Institute space). Studies not fulfilling these requirements were excluded. No age, gender or treatment restrictions were applied.

Additionally, facial affect processing studies were included if they used human facial identities as stimuli in both active and control condition (as opposed to geometric shapes) and were excluded if they used facial stimuli to investigate processes not directly involved in emotional processing, such as working memory or attention. As the majority of FER studies used negatively valence facial expressions (anger, fear, sadness) we focused exclusively on these to minimize heterogeneity due to valence [33].

Information extracted from each included: a) foci of task-related neural changes contrasting an active and control condition between either patient group (ASD or SZ) and healthy controls b) a description of each task and the selected contrast c) clinical Information concerning age, gender, diagnosis, symptom scales, illness duration and treatment, and matching of the healthy control group.

### Quantitative meta-analytical voxel-based procedure

We used one contrast from each study as shown in Tables 1 and 2, and we accepted results as significant based on the threshold employed in the original studies.

**Table 1:** Details of studies included in the meta-analysis for facial emotion recognition tasks.

Reference	Patients/Controls	Task	Contrast used	Matching
**Autism Spectrum Disorders**				
Hall et al, 2010 [60]	12/12	Implicit	Anxious>Neutral faces	Gender, age, non-verbal IQ
Monk et al, 2010 [61]	12/12	Implicit	Sad>Neutral faces	Gender, age, handedness, IQ
Deeley et al, 2007 [62]	9/9	Implicit	Fear>Neutral faces	Gender, age, verbal IQ
Ashwin et al, 2007 [63]	13/13	Implicit	Fear>Scrambled faces	Gender, age, handedness
Critchley et al, 2000 [64]	9/9	Implicit and Explicit	Angry>Neutral faces	Gender, age, IQ
**Schizophrenia**				
Rauch et al, 2010 [65]	12/12	Implicit	Sad>Neutral faces	Age, handedness
Dowd et al, 2010 [66]	32/40	Explicit valence rating task	Negative>Neutral	Gender, age, handedness, parental education
Seiferth et al, 2009 [67]	12/12	Explicit labeling task	Fearful>Neutral faces	Gender, age, handedness, parental education
Reske et al, 2009 [68]	18/18	Emotion discrimination task	Sad>Neutral faces	Gender, age, parental education
Michalopoulou et al, 2008 [69]	11/9	Implicit	Fearful>Neutral faces	Age, handedness, education
Hall et al, 2008 [70]	19/24	Implicit	Fearful>Neutral faces	Gender, age, IQ
Williams et al, 2007 [71] (paranoid patients)	13/13	Implicit	Fearful>Neutral faces	Gender, age, handedness, IQ
Williams et al, 2007 [71] (non-paranoid patients)	14/13	Implicit	Fearful>Neutral faces	Gender, age, handedness, IQ
Gur et al, 2007 [72]	16/17	Emotion discrimination task	Fearful>Neutral faces	Gender, handedness, parental education
Das et al, 2007 [73]	14/14	Implicit and Explicit	Fearful>Neutral	Gender, age, handedness
Surguladze et al, 2006 [74]	15/11	Implicit	Fearful>Neutral faces	Gender, age, handedness, education
Holt et al, 2006 [75]	15/16	Implicit	Fearful>Neutral	Gender, age, education, parental education
Kosaka et al, 2002 [76]	12/12	Emotional intensity judgment task	Negative>Neutral faces	Gender, age, handedness

**Table 2:** Clinical description of patients with Autism Spectrum Disorders included in facial emotion recognition studies.

Reference	Mean age (SD) Patients/Controls	Gender (% male) Patients/Controls	Symptom Scales Mean score (SD)	Diagnosis/Recruitment/Illness Duration	Medication Dose (mg): mean (SD)*
Hall et al, 2010 [60]	31.8/32	100%/100%	ADOS (Communication): 5.22 (1.39) ADOS (Social interaction): 10.33 (2.73)	HFA, Asperger, PDD-NOS	No information provided
Monk et al, 2010 [61]	26/27	100%/100%	ADI-R/ADOS	HFA (n = 7), Asperger(n = 2), PDD-NOS (n = 3)	SSRI (n = 5), stimulants (n = 4), NLP (n = 2), TCA (n = 1),BZD (n = 1)
Deeley et al, 2007 [62]	34 (10)/27(5)	100%/100%	ADI-R/ADOS	Asperger	Unmedicated
Ashwin et al, 2007 [63]	31/25	100%/100%	Autism Quotient: 35.6 (6.3)	HFA/Asperger	Unmedicated
Critchley et al, 2000 [64]	37(7)/27(7)	100%/100%	ADI-R	HFA (n = 2), Asperger (n = 7)	No information provided

ADI-R = Autism Diagnostic Interview-Revised [77]; ADOS = Autism Diagnostic Observation Schedule [78]; BZD = benzodiazepine; HFA = High Functioning Autism; PDD-NOS = Pervasive Developmental Disorder Not Otherwise Specified; NLP = neuroleptics; SSRI = selective serotonin reuptake inhibitor; TCA = tricyclic antidepressant.

### Activation Likelihood Estimation procedure

Coordinates from FER and ToM studies were analysed separately following the Activation Likelihood Estimation (ALE) technique implemented in GingerALE 2.0.4 (http://brainmap.org/Ale). This version uses a random effect model and weighting for sample size of the original studies [34]. Coordinates of the foci of activation reported in the original studies were transformed into Talairach space using the Lancaster transform (icbm2tal tool) in GingerALE. For each study, peaks were modelled as the centre of a 3D Gaussian distribution and a modelled activation (MA) map was then computed. ALE scores from the convergent MA maps were then calculated on a voxel-by-voxel basis to test for convergent (random-effects) rather than study specific foci (fixed-effects). All ALE data processing was performed using the BrainMap Search and View software (http://brainmap.org). The threshold of statistical significance was set at p<0.05, with False Discovery Rate (FDR) correction for multiple comparisons and a minimum cluster size of 80 mm^3^. Each ALE map was imported into Mango (http://ric.uthscsa.edu/mango) and overlaid on an anatomical Template (http://www.brainmap.org/ale/colin1.1.nii) for representation purposes. Significant clusters were manually localized and Brodmann areas (BA) were identified using the standard Talairach and Tournoux [35] stereotactic anatomic brain atlas. Finally, in order to compare diagnostic groups, the ALE Maps for SZ and ASD for each experimental design (e.g. ALE map for ASD FER vs ALE map for SZ FER) were directly contrasted using subtraction meta-analysis procedure implemented in GingerALE version 1.1 [34]. Regions in which the two diagnostic groups differed were defined at p<0.05 with FDR correction and a minimum cluster size of 200 mm^3^.

### Demographic data analysis

Student *t* tests (two-tailed) or chi-square tests were used to compare the distribution of continuous and categorical demographic variables between the two groups. Only variables which displayed group differences were subjected to voxel-wise meta-regression analyses in order to examine their effect on the ALE results.

## Results

The initial search returned 415 citations. Of these, 337 studies were discarded after reviewing the abstracts while the full text of the remaining 76 citations was examined in more detail. Thirty three studies fulfilled the inclusion criteria and were included in the meta-analysis. Details are shown in Tables 1 and 2.

### Facial emotion recognition

We identified 16 FER studies in ASD and 33 in SZ, of which 5 and 12 respectively were used in the ALE analysis. The total sample comprised 55 ASD and 203 SZ patients and 253 healthy controls (HC) (Table 1). Demographic details for all participants and clinical information of ASD and SZ patients are shown in Tables 2 and 3. Mean age did not differ between diagnostic groups (ASD: mean = 31.96, SD = 4.06; SZ: mean = 31.41, SD = 8.47; t = 0.13, df = 16, p = 0.89,), but there was an over-representation of males in the ASD studies (ASD: 100% males; SZ: 74.93% males; chi-square = 20.4, df = 1, p<0.001).

**Table 3:** Clinical description of patients with Schizophrenia included in facial emotion recognition studies.

Reference	Age Mean (SD) Patients/Controls	Gender (% male) Patients/Controls	Symptom Scales Mean score (SD)	Diagnosis/Recruitment/Illness Duration	Medication Dose (mg): mean (SD)*
Rauch et al, 2010 [65]	27.7 (7.5)/26.9 (6.1)	58.3%/75%	PANSS Positive: 14.4 (3.1); PANSS Negative: 18.9 (5.2); PANSS General: 34.9 (5.9)	Inpatients	NLP: 902.1 (603.2)
Dowd et al, 2010 [66]	36.25 (10.85)/36.8 (8.99)	65.2%/65%	SAPS: 1.83 (.93); SANS: 1.81 (1.37)	Schizophrenia/schizoaffective disorder outpatients. Duration of illness: 17.73 years (11.25).	NLP: 452.20 (369.60)
Seiferth et al, 2009 [67]	17.8 (1.4)/17.9 (1.5)	100%/100%	PANSS Positive: 16.2 (8.1); PANSS Negative: 13.3 (8.9)	Early onset schizophrenia, Mean duration of illness: 38 weeks	NLP: 231 (111)
Reske et al, 2009 [68]	31.94 (6.41)/31.94 (6.03)	55.5%/55.5%	PANSS Positive: 8 (1.14); PANSS Negative: 13.61 (4.47); PANSS total: 23.11 (3.94)	First episode psychosis, inpatients	Double blind treatment with haloperidol (mean dose 2.56mg/d) or risperidone (mean dose 2.23 mg/d) for 6 to 8 weeks
Michalopoulou et al, 2008 [69]	35 (9)/32 (6)	81.2%/55.5%	PANSS positive: 16 (6.72); PANSS negative: 13.91 (5.54); PANSS total: 58.91 (17.72)	Outpatients. Duration of illness: 12 years (9)	NLP: 523 (455)
Hall et al, 2008 [70]	37.7 (8.4)/35.1 (9.7)	63.15%/66.6%	PANSS Positive: 12.3 (4.5)	Inpatient and outpatients. OPCRIT criteria	NLP: 496 (377)
Williams et al, 2007 [71] (paranoid patients)	26.9 (9.1)/25.1 (8.1)	61.5%/63%	PANSS positive (delusions):5.2 (1.2); (excitement): 3.7 (0.6)	Duration of illness: 5.6 years (4.6)	NLP: 375.1 (290.6)
Williams et al, 2007 [71] (non-paranoid patients)	27.8 (10.4)/25.1 (8.1)	64.3%/63%	PANSS Positive (delusions): 2.1 (1.1); 1.6 (0.8)	Duration of illness: 5.6 years (4.6)	NLP: 339.3 (240.3)
Gur et al, 2007 [72]	30.1 (6.5)/25 (3.9)	75%/70.6%	SAPS: 1.4 (0.6); SANS: 1.3 (0.9)	Duration of illness: 9.6 years (7.1), hospitalizations: 3.6 (4.1)	Untreated (n = 1); First generation NLP (542 mg/day (292)) (n = 2); Olanzapine (18.2 mg/day (2.8)) (n = 11); Both (n = 2).
Das et al, 2007[73]	20.4 (3.3)/23.1 (5.9)	100%/100%	PANSS Positive: 16.07 (7.24); PANSS Negative: 21.14 (7.9)	First episode psychosis, Duration of illness: 1.21 years (1.2)	Unmedicated (n = 5); Atypical NLP, 393.4 (213.2) (n = 9)
Surguladze et al, 2006 [74]	43.1 (8.8)/36.8 (10.6)	100%/100%	SAPS: 25.1 (23.9); SANS: 27.9 (14.6)	Duration of illness: 19.9 years (10.5), hospitalizations 5.7 (5.4)	NLP: 487.1 (range: 100–1200 mg/day)
Holt et al, 2006 [75]	47.7 (7.1)/48.2 (9.6)	100%/100%	PANSS Positive and Negative Syndrome Scale: 59.8 (10.3)	Outpatients. Duration of illness: 21.6 years (9.6)	NLP: 424.5 (268.8)
Kosaka et al, 2002 [76]	26 (4.5)/24.4 (2.4)	50%/50%	PANSS Positive: 11-3 (4.6); PANSS Negative: 16.3 (4.5); PANSS General: 28.8 (7.3)	Outpatients and inpatients. Duration of illness: 3.8 years (3.5)	NLP: 322.0 mg (264.1) Unmedicated (n = 2)

Unless otherwise specified, neuroleptic dose expressed in chlorpromazine equivalents; NLP = neuroleptics; PANSS = Positive and Negative Syndrome Scale for Schizophrenia [79]; SAPS = Scale for the Assessment of Positive Symptoms [80]; SANS = Scale for the Assessment of Negative Symptoms [81];

#### Autism spectrum disorders vs. healthy controls

**Figure 1:**
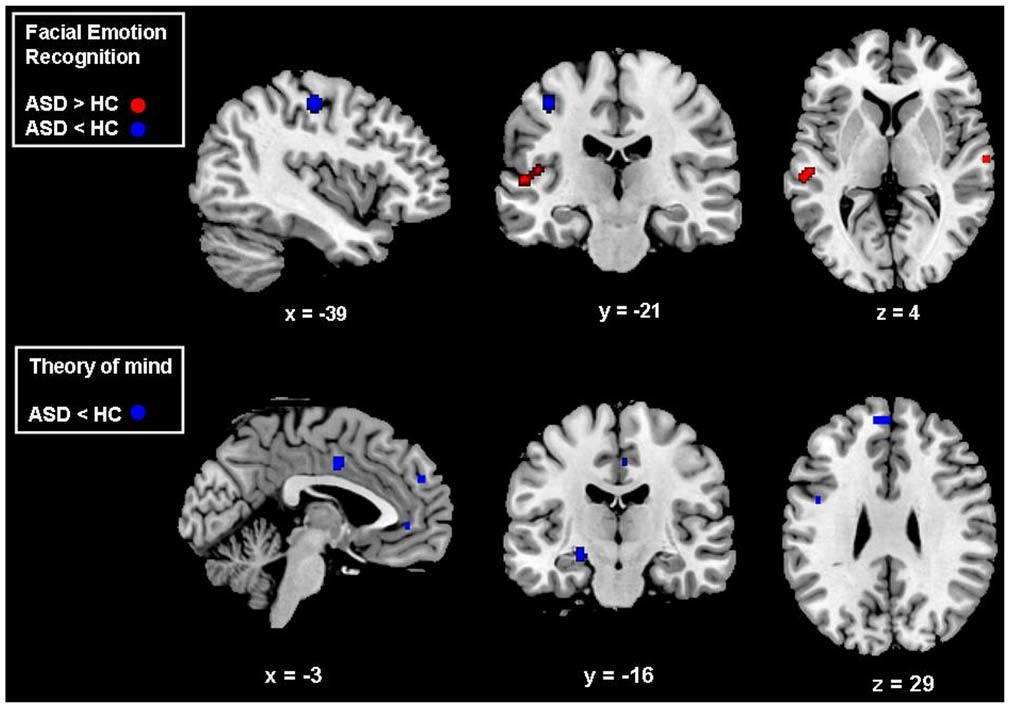
Activation likelihood estimation maps comparing autism spectrum disorder subjects with healthy controls. Clusters of relative underactivation or overactivation in ASD are shown in blue and red respectively; numbers represent the sagittal (x), coronal (y) and axial (z) coordinates of each slice in Talairach space. p<0.05 False Rate Discovery corrected for multiple comparisons. ASD = Autism Spectrum Disorders, HC = Healthy Controls.

During the emotional faces>neutral faces contrast (ASD = 34, HC = 34, foci = 9), ASD patients compared to HC showed increased activation in temporal regions near the STS and decreased activation in the primary somatosensory cortex, within the postcentral gyrus. The corresponding anatomical regions and peak ALE maxima are show in Table 4 and Figure 1.

**Table 4:** Activation likelihood estimation results for facial emotion recognition in patients with schizophrenia or autism spectrum disorders compared to healthy controls.

Brain region	Gyrus	BA	Laterality	Site of maximum ALE*			Volume (mm^3^)	Maximum ALE value
				x	y	z		
**Autism spectrum disorders**								
*ASD>HC*								
Temporal	Superior Temporal	42/22	Left	−56	−24	6	424	0.01067
*ASD<HC*								
Parietal	Postcentral	3	Left	−40	−20	50	320	0.01286
**Schizophrenia**								
*SZ>HC*								
No significant clusters	-	-	-	-	-	-	-	-
*SZ<HC*								
Frontal	Inferior Frontal	47	Right	36	18	−18	80	0.09672
Limbic	Posterior Cingulate	23	Right	4	−26	24	80	0.00872
Limbic	Amygdala		Left	−22	−2	−12	96	0.01012
Occipitopemporal	Fusiform	19	Right	28	−74	−10	200	0.01012
Thalamus	Dorsomedial		Left	−2	−12	12	208	0.01018

*x = sagittal, y = coronal, z = axial coordinates according to Talairach and Tournoux; BA = Brodmann Area; ALE = Activation Likelihood Estimation, p<0.05 False Rate Discovery corrected for multiple comparisons. SZ = Schizophrenia, ASD = Autism Spectrum Disorders, HC = Healthy Controls.

#### Schizophrenia vs. healthy controls

**Figure 2:**
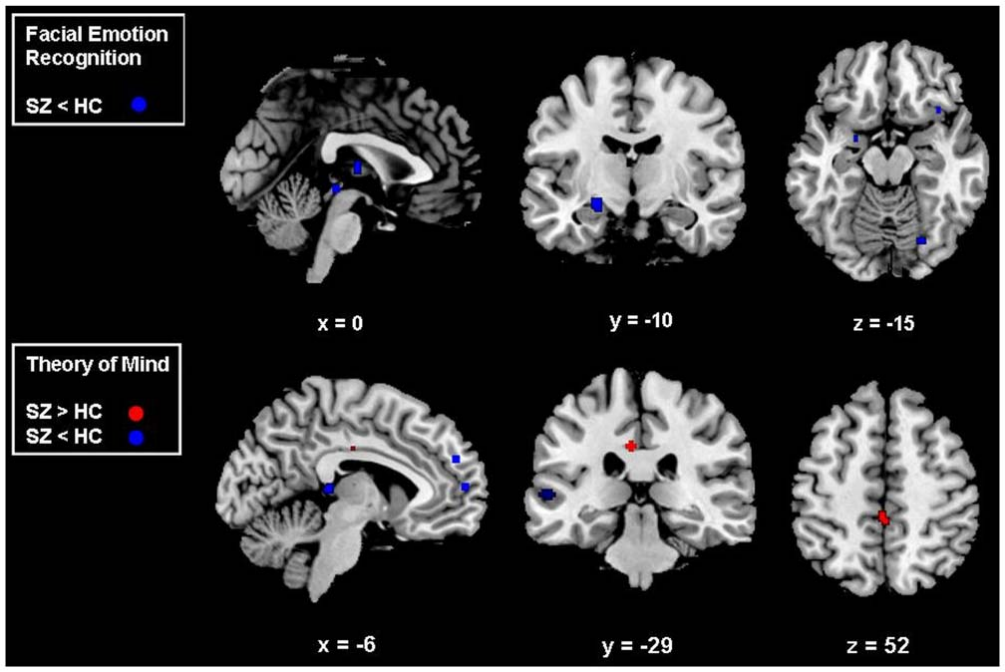
Activation likelihood estimation maps comparing schizophrenia patients with healthy controls. Clusters of relative underactivation or overactivation in SZ are shown in blue and red respectively; numbers represent the sagittal (x), coronal (y) and axial (z) coordinates of each slice in Talairach space. p<0.05 False Rate Discovery corrected for multiple comparisons. SZ = Schizophrenia, HC = Healthy Controls.

During the same contrast (SZ = 77, HC = 77, foci = 16), SZ patients compared to HC showed decreased activation in the ventrolateral PFC, posterior cingulate cortex, amygdala, occipito-temporal regions (encompassing the fusiform gyrus) and thalamus (Table 4, Figure 2).

#### Comparison of autism spectrum disorders and schizophrenia

**Figure 3:**
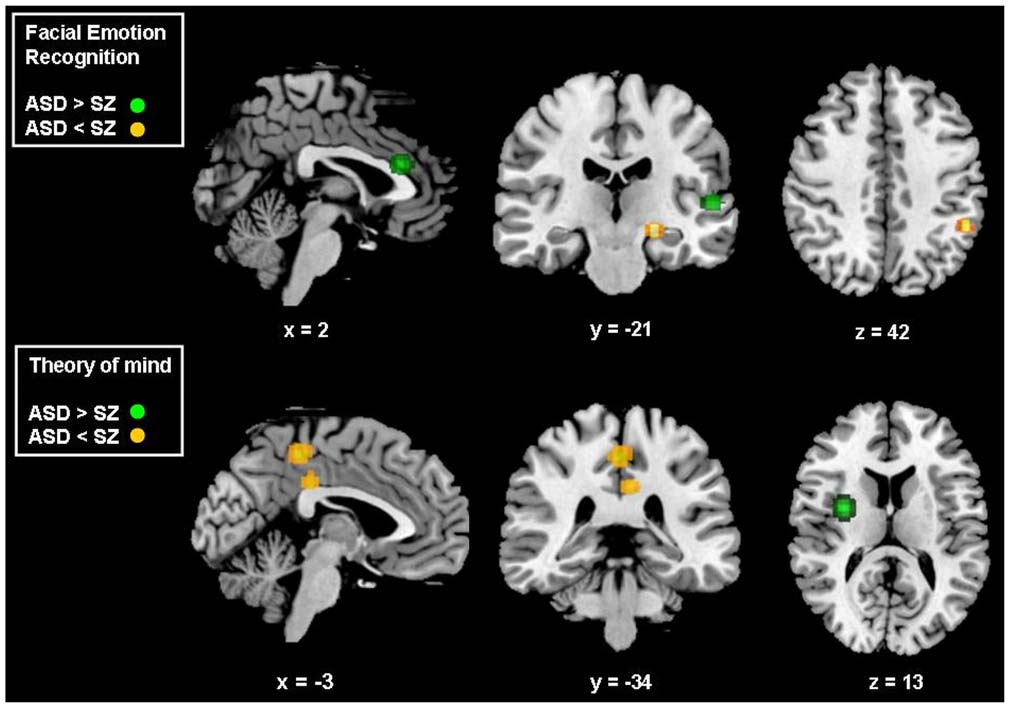
Activation likelihood estimation maps comparing autism spectrum disorder with schizophrenia patients. Clusters of relative overactivation and underactivation in ASD are shown in green and yellow respectively; numbers represent the sagittal (x), coronal (y) and axial (z) coordinates of each slice in Talairach space. p<0.05 False Rate Discovery corrected for multiple comparisons. ASD = Autism Spectrum Disorders, SZ = Schizophrenia.

ASD subjects were significantly more likely than SZ to engage temporal regions near the STS and in the anterior and posterior cingulate cortex. In the inverse contrast, SZ patients expressed greater likelihood than ASD to engage the ventrolateral PFC, the parahippocampal gyrus and regions within the TPJ, inferior occipital gyrus and the cerebellum (Table 5, figure 3).

**Table 5:** Activation likelihood estimation results for facial emotion recognition comparison of schizophrenia and autism spectrum disorders.

Brain region	Gyrus	BA	Laterality	Site of maximum ALE			Volume (mm^3^)	Maximum ALE value
				x	y	z		
***Autism Spectrum Disorders>Schizophrenia***								
Limbic	Anterior Cingulate	24	Left	0	26	20	392	0.00652
Limbic	Anterior Cingulate	32	Right	10	34	20	376	0.00652
Limbic	Posterior Cingulate	30	Left	−20	−62	4	320	0.00645
Temporal	Superior Temporal	42/22	Left	−56	−24	6	1824	0.00884
Temporal	Superior Temporal	22	Right	40	−48	14	432	0.00663
***Schizophrenia>Autism Spectrum Disorders***								
Frontal	Inferior Frontal	47	Left	−36	28	2	392	0.00646
Limbic	Parahippocampus	28	Left	−22	−22	−10	392	0.00629
Parietal	Inferior Parietal	40	Left	−50	−44	−40	360	0.00647
Occipital	Inferior Occipital	18	Right	32	−84	−4	304	0.00646
Cerebellum	Culmen	-	Left	−30	−46	−20	352	0.00654
Cerebellum	Culmen	-	Right	32	−44	−18	304	0.00639
Cerebellum	Declive	-	Left	−30	−76	−20	384	0.00651
Cerebellum	Declive	-	Right	26	68	−14	376	0.00651

x = sagittal, y = coronal, z = axial coordinates according to Talairach and Tournoux. BA = Brodmann Area; ALE = Activation Likelihood Estimation, p<0.05 False Rate Discovery corrected for multiple comparisons.

### Theory of mind

Our search identified 10 studies exploring ToM related processes in ASD subjects and 17 in SZ patients, of which 7 and 9 respectively fulfilled inclusion criteria (Table 6). The total analysis encompassed 91 ASD and 133 SZ patients, and 239 HC subjects. Demographic details for all participants and clinical information corresponding to ASD and SZ patients are shown in Tables 7 and 8. ASD subjects were significantly younger than SZ (ASD: mean = 18.55, SD = 7.03; SZ: mean = 31.36, SD = 4.08; t = 4.1, df = 13, p = .001), and included a higher percentage of males (ASD: mean = 95.2%,; SZ: mean = 63.0%, chi-quare = 47.01, df = 1, p<0.001).

**Table 6:** Details of studies included in the meta-analysis for theory of mind tasks.

Reference	Patients/Controls	Task	Contrast used	Matching
**Autism spectrum disorders**				
Lombardo et al, 2010 [82]	23/23	Judgment of self or other physical or mental states	Other mentalising>Self mentalising	Gender, age, IQ, handedness
Uddin et al, 2008 [83]	8/12	Self face recognition paradigm	Self>Rest	Gender, age, IQ, handedness
Wang et al, 2007 [84]	18/18	Judgment of irony	Irony>No Irony conditions	Gender, age, handedness
Dapretto et al, 2006 [85]	10/10	Imitation of facial expressions	Imitation of facial expressions>Passive observation	Gender, age, IQ
Williams et al, 2006 [86]	16/15	Imitation of facial expression	Imitation>Observation of animated facial expressions	Gender, age, IQ, handedness
Pelphrey et al, 2005 [87]	10/9	Judgment of congruency of eye gaze shift	Incongruent>Congruent gaze shift	Gender, age, IQ, handedness
Baron-Cohen et al, 1999 [53]	6/12	Judgment of mental states from pictures of eyes	Judgment of mental state>Judgment of gender	Gender, age, IQ, handedness, socioeconomic status, education
**Schizophrenia**				
Park et al, 2011 [88]	14/15	Listening and viewing of self or other referential vignettes	Other referential>Neutral	Gender, age, handedness, education
Lee et al, 2010 [89]	15/18	Empathic story completion task	Empathic>Factual	Gender, age, verbal IQ, handedness, education
Holt et al, 2010 [90]	17/18	Self or other referential affect labeling, and perceptual processing of adjectives	Other referential>Perceptual processing	Gender, age, IQ, handedness, ethnicity, parental socio-economic status
Walter et al, 2009 [48]	12/12	Intentional story completion task	Intentional>Factual causality	Gender, age, handedness, education
Benedetti et al, 2009 [91]	24/20	Judgment of intentions or empathy	Intention and empathy>Physical causality	Gender, age, handedness
Dollfus et al, 2008 [92]	23/23	Judgment of corruption	Judgment of corruption>Factual	Gender, age, handedness, education
Brune et al, 2008 [93]	9/13	Judgments of cooperation or deceit	Mentalising judgment>Factual judgment	Gender, age, handedness
Lee et al, 2006 [94]	14/14	Judgment of empathy	Empathic>Factual judgments	Gender, age, handedness, IQ
Russell et al, 2000 [95]	5/7	Judgment of mental states from pictures of eyes	Judgment of mental state>Judgment of gender	Gender, age, handedness, education

**Table 7:** Clinical description of patients with Autism Spectrum Disorders included in theory of mind studies.

Reference	Mean age (SD) Patients/Controls	Gender (% male) Patients/Controls	Symptom Scales Mean scores (SD)	Diagnosis/Recruitment/Illness Duration	Medication Dose (mg): mean (SD)*
Lombardo et al, 2010 [82]	27.97 (6.10)/26.59 (7.04)	100%	ADI-R: (Social): 18.07 (5.07), (Communication): 15.17 (4.24), (Rep): 5.97 (2.76) AQ (ASD): 32.59 (8.20); AQ (HC): 15.24 (6.89)	Asperger Syndrome	No information provided
Uddin et al, 2008 [83]	13.19 (2.61)/12.23 (2.1)	100%	ADOS, ADI-R	HFA	None
Wang et al, 2007 [84]	11.8 (2.9)/11.8 (1.9)	100%	ADOS, ADI-R, Social Responsiveness Scale	HFA	Medicated (SSRIs, stimulants, atypical NLP) (n = 7)
Dapretto et al, 2006 [85]	12.05 (2.5)/12.38 (2.2)	100%	ADOS (Social) 8.5 (3.0); ADI (Social): 20.3 (4.9)	HFA	No information provided
Williams et al, 2006 [86]	15.4 (2.24)/15.5 (1.60)	100%/100%	ADOS, ADI-R	HFA	Methylphenidate (n = 2), clonidine, dexamphetamine and melatonin (n = 1)
Pelphrey et al, 2005 [87]	23.2 (9.9)/23.4 (5.8)	90%/89%	ADOS, ADI-R	HFA	No information provided
Baron-Cohen et al, 1999 [53]	26.3 (2.1)/25.5 (2.8)	67%/50%	DSM-IV, ICD-10	HFA	No information provided

ADI-R = Autism Diagnostic Interview-Revised [77]; ADOS = Autism Diagnostic Observation Schedule [78]; AQ = Autism Spectrum Quotient [96]; DSM-IV = Diagnostic and Statistical Manual of Mental Disorders, 4th Edition; HFA = High Functioning Autism; ICD-10 = International Classification of Diseases, 10^th^ Revision; NLP = neuroleptics; SSRI = selective serotonin reuptake inhibitor.

**Table 8:** Clinical description of patients with Schizophrenia included in theory of mind studies.

Reference	Mean age (SD) Patients/Controls	Gender (% male) Patients/Controls	Symptom Scales Mean scores (SD)	Diagnosis/Recruitment/Illness Duration	Medication Dose (mg): mean (SD)*
Lee et al, 2010 [89]	26.0 (4.3)/25.8 (2.2)	47%/50%	PANSS Positive = 13.1 (5.1); PANSS Negative = 15.4 (4.1); PANSS Total = 64.4 (11.0)	Stable outpatients; Duration of illness: 4.6 years (3.4)	422.1 (23.7); Olanzapine (n = 4), aripirazole (n = 4), risperidone (n = 2), amisulpride (n = 2), clozapine (n = 1), perphenacine (n = 1), ziprasidone (n = 1)
Holt et al, 2010 [90]	35.9 (13.7)/40.0 (12.5)	65%/67%	PANSS Positive = 11.7 (3.6); PANSS Negative = 11.4 (3.2); PANSS General = 24.2 (5.2); PANSS Total = 47.3 (10.0)	Stable outpatients; Duration of illness: 13.2 years (12.8)	NLP 374.1 (364.0)
Walter et al, 2009 [48]	29.5 (6.9)/24.7 (2.6)	50%/50%	PANSS Positive = 17.75 (5.06); PANSS Negative = 19.41 (3.96); PANSS Total = 73.75 (11.02); Brief Psychiatric Rating Scale = 47.18 (10.6)	Inpatients; Duration of illness: 6.3 years (5.2)	No information provided
Benedetti et al, 2009 [91]	37.2 (10.23)/35.1 (9.95)	58%/5%	PANSS Positive = 16 (4.58); PANSS Negative = 21.66 (5.42); PANSS Total = 72.57 (14.49)	Stable outpatients; SZ subtypes: undifferentiated (n = 9), paranoid (n = 13), disorganized (n = 2); Duration of illness: 12.7 years (6.96)	357.81 (256.17). Monotherapy, stable doses for prior 3 months. Clozapine (n = 9), risperidone (n = 11), aripiprazole (n = 2), haloperidol (n = 2).
Dollfus et al, 2008 [92]	29.9 (7.9)/30.0 (8.6)	78%/78%	PANSS Positive = 11.7 (4.5); PANSS Negative = 13.1 (5.0); PANSS Total = 49.5 (14.8)	Stable outpatients;. Duration of illness: 7.6 years (7.2)	NLP: 315.5 (256.2)
Brune et al, 2008 [93]	27.89(6.6)/26.46(5.30)	33%/31%	PANSS Positive = 16.67 (5.75); PANSS Negative = 15.67 (8.87)	First Episode (n = 6), recurrent episodes (n = 3); Duration of illness: 3.0 years (4.23)	NLP:244.44 (173)
Lee et al, 2006 [94]	31.7(7.3)/30.5(8.8)	93%/93%	SAPS = 2.4 (2.0); SANS = 5.4 (3.0)	Outpatients; Duration of illness: 9.8 years (5.4)	406.4 (205.6); Clozapine (n = 5), olanzapine (n = 4), risperidone (n = 1), typical NLP (n = 4).
Russell et al, 2000 [95]	36 (9)/40(11)	100%/100%	DSM-IV criteria	Duration of illness: 13 years (7)	Receiving NLP

Unless otherwise specified neuroleptic dose expressed in chlorpromazine equivalents; duration of illness is presented as mean (standard deviation); DSM-IV = Diagnostic and Statistical Manual of Mental Disorders, 4th Edition; NLP = neuroleptics; PANSS = Positive and Negative Syndrome Scale for Schizophrenia [79]; SAPS = Scale for the Assessment of Positive Symptoms [80]; SANS = Scale for the Assessment of Negative Symptoms [81].

#### Autism spectrum disorders vs. healthy controls

In the contrast ToM>control condition (ASD = 88, HC = 98, foci = 33), ASD subjects compared to HC showed decreased activation in six clusters located in the medial prefrontal and in the anterior cingulate cortex, the amygdala and in primary and secondary somatosensory areas in the precentral gyrus, the STS, and in the TPJ within the inferior parietal lobule (Table 9, Figure 1).

**Table 9:** Activation likelihood estimation results for theory of mind in patients with schizophrenia or autism spectrum disorders compared to healthy controls.

Brain Region	Gyrus	BA	Laterality	Site of maximum ALE			Volume (mm^3^)	Maximum ALE value
				x	y	z		
**Autism Spectrum Disorders**								
*Autism Spectrum Disorder>Healthy Controls*								
No significant clusters	-	-	-	-	-	-	-	-
*Autism Spectrum Disorder<Healthy Controls*								
Frontal	Medial Frontal	9/10	Left	−2	52	28	176	0.01022
Frontal	Precentral	44	Right	54	8	8	96	0.00939
Limbic	Anterior Cingulate	24	Left	−2	−2	40	248	0.01126
Limbic	Amygdala		Left	−22	−8	−8	272	0.01309
Temporal	Middle Temporal	21	Left	−56	−36	0	176	0.01038
Parietal	Inferior Parietal	40	Left	−38	−32	48	344	0.01117
**Schizophrenia**								
*Schizophrenia>Healthy Controls*								
Parietal	Paracentral Lobule	5	Right	2	−38	54	176	0.00946
Limbic	Posterior Cingulate	23	Left	0	−16	24	88	0.00971
*Schizophrenia>Healthy Controls*								
Frontal	Medial Frontal	9/10	Left	−6	50	26	64	0.00922
Limbic	Posterior Cingulate	23	Right	10	−32	22	232	0.01019
Temporal	Middle Temporal	22	Left	−58	−36	4	120	0.00978
Thalamus	Pulvinar		Left	−6	−32	8	272	0.01134

x = sagittal, y = coronal, z = axial coordinates according to Talairach and Tournoux; BA = Brodmann Area; ALE = Activation Likelihood Estimation, p<0.05 False Rate Discovery corrected for multiple comparisons.

#### Schizophrenia vs. healthy controls

In the same contrast (SZ = 133, HC = 140, foci = 43) SZ patients compared to HC showed (a) increased activation in the posterior cingulate cortex and within the somatosensory cortices in the paracentral lobule, and superior temporal gyrus and (b) decreased activation in the medial prefrontal PFC, middle temporal gyrus and the thalamus (Table 9, Figure 2).

#### Comparison of autism spectrum disorders and schizophrenia

ASD was associated with greater likelihood of activation in the insula compared to SZ (Table 10). In the inverse contrast, SZ was associated with greater likelihood of engagement in regions within the medial PFC and somatosensory areas in the paracentral gyrus, and in the posterior cingulate cortex (Table 8; Figure 3). The effect size of signal differences in the medial PFC region was positively correlated with age (peak coordinates = −26, 50, 2; r^2^ = 0.28; p = 0.001; voxels = 59).

**Table 10:** Activation likelihood estimation results for theory of mind tasks comparison of schizophrenia and autism spectrum disorders.

Brain region	Gyrus	BA	Laterality	Site of maximum ALE			Volume (mm^3^)	Maximum ALE value
				x	y	z		
***Autism Spectrum Disorders>Schizophrenia***								
Limbic	Insula	13	Right	32	−2	12	200	0.00636
***Schizophrenia>Autism Spectrum Disorders***								
Frontal	Medial Frontal	10	Right	8	60	4	168	0.00627
Frontal	Paracentral Lobule	5	Left	0	−36	52	656	0.00784
Limbic	Posterior Cingulate	23	Left	0	−16	24	624	0.00706
Limbic	Posterior Cingulate	31	Left	−6	−30	34	200	0.00665

x = sagittal, y = coronal, z = axial coordinates according to Talairach and Tournoux; BA = Brodmann Area; ALE = Activation Likelihood Estimation, p<0.05 False Rate Discovery corrected for multiple comparisons.

### Examination of confounding factors

#### Age

The effect size of signal differences in the medial PFC region was positively correlated with age (peak coordinates = −26, 50, 2; r^2^ = 0.28; p = 0.001; voxels = 59). This would suggest that some of the differences observed between ASD and SZ in this area may be also modulated by age.

#### Antipsychotic medication

Details of pharmacological treatments are displayed in tables 2 and 4. The mean dose of antipsychotic drugs was converted into chlorpromazine equivalent doses. Studies providing information on the mean daily chlorpromazine equivalent dose of antipsychotics for their sample were included in voxel-wise meta-regressions exploring the relationship between dose and magnitude of ALE scores. Antipsychotic medications were found to have a normalizing effect on amygdala involvement during FER. The effect size of signal differences in the amygdala was negatively correlated with dose of antipsychotics (peak coordinates: [30, −2, −18]; r^2^ = 0.48; p = 0.001; number of voxels = 45), and therefore it is unlikely that the differences observed between ASD and SZ are due to the effects of antipsychotic medication. With regards to ToM, we also found an ameliorative effect of medication on the activation in the superior temporal gyrus (peak voxels: [46, 10, −24]; r^2^ = 0.48; p = 0.001; number of voxels = 237; and [44, 16, 12]; r^2^ = 0.27; p = 0.001; number of voxels = 171), which we also interpret as unlikely to be the source of the group differences.

## Discussion

We used quantitative analysis to explore the neural correlates of social cognition during facial emotion recognition (FER) and theory of mind (ToM) tasks in ASD and SZ. Differential effects of task and diagnosis were noted in the distribution and direction of the findings. Our key findings can be summarised as follows: (a) Both SZ and ASD patients showed reduced engagement in medial PFC regions, with the extent and degree of deficit being greater in ASD; conversely, ventrolateral PFC disruption was associated mostly with SZ, (b) SZ was associated with hyperactivation in somatosensory cortices, while ASD subjects showed reduced activity in these regions, (c) amygdala hypoactivation was observed in SZ patients during facial emotion processing and in ASD during more complex ToM tasks, (d) both SZ and ASD were associated with decreased engagement of regions around the STS during ToM tasks, but when processing facial affect, overactivation in these regions was noted in ASD, (e) reduced thalamic engagement was uniquely associated with SZ.

### Prefrontal and midline cortical involvement

We found both similarities and differences in prefrontal engagement in ASD and SZ. During FER tasks, SZ patients were significantly more likely than ASD to engage the ventrolateral PFC, but the degree of activation was less than in controls. Both SZ and ASD were associated with hypoactivation in medial PFC regions during ToM tasks, although the deficits were quantitatively greater in ASD and extended more posteriorly into the anterior and posterior cingulate cortex.

The role of the ventrolateral PFC in social cognition has begun to be more fully appreciated as it is involved in multiple tasks of social cognition [17]. This region is thought to modulate the influence of emotional stimuli on cognition with respect to contextually (or socially) appropriate behaviour [12], [18], [19], [36]. Our findings of hypoactivation in SZ patients in this region reinforce the results of Leitman and colleagues [37] who demonstrated reduced contextual modulation of the ventrolateral PFC in SZ in connection to impaired affective appraisal. In contrast, activation in the medial prefrontal cortex is thought to underlie emotion generation, particularly when assessing self-relevant attributes or emotional awareness [38]–[40]. Therefore our results imply that reduced PFC engagement in connection to the social appropriateness of behaviour may be more relevant to SZ, while in both SZ and ASD, reduced medial PFC engagement may contribute to abnormalities in conscious awareness of the emotional states of others.

### Somato-sensory cortices

Activation in primary and secondary somato-sensory cortices was reduced in ASD, in comparison to controls and SZ patients, during both FER and ToM tasks. Engagement of somato-sensory cortices is thought to invoke (“mirror”) bodily states associated with pertinent emotions or other internal states, thus facilitating their recognition in oneself or others [11], [41], [42]. Our findings in ASD are consistent with reports of abnormal cortical thickness and grey matter volume patterns in the corresponding anatomical regions [43], [44] and lend further support to the notion that ASD is associated with dysfunction in invoking “mirroring” mechanisms when processing social stimuli [45]. Our evidence of increased involvement of somato-sensory regions during social cognitive tasks in SZ adds to similar findings [46], [47], and may contribute to the over-attribution of agency and “hypermentalization” described in schizophrenia [48], [49].

### Amygdala

Decreased engagement of the amygdala was observed in SZ patients during FER tasks, which confirms findings from earlier meta-analytic studies [28], [50]. However, Anticevic et al [50] have reported that in SZ there is increased amygdala activation to neutral stimuli, which may have reduced the contrast between emotional and neutral conditions employed here. In ASD subjects, amygdala hypoactivation was observed during ToM tasks, in line with previous reports of significant amygdala pathology in autism [51]–[53]. Our results suggest that both SZ and ASD show deficits in engaging the amygdala when processing social stimuli, but this observation may depend on stimulus type. In SZ, reduced amygdala activation was associated with tasks of attribution of affective states, while in ASD it was mostly seen during tasks of epistemic and intentional attributions. We can only speculate as to the underlying mechanisms of this stimulus-specific dissociation of amygdala engagement in SZ and ASD.

The amygdala is not a homogeneous structure but is comprised of 13 nuclei. The functional specificity of the amygdala nuclei remains unclear, since selective lesions in humans have not been reported. Despite evidence that damage to basal and lateral nuclei may be more directly linked to recognition of facial expressions [54], [55] it is not known whether there is regional specialisation within the amygdala for processing other social stimuli. However, our data suggest that the possibility of disease specific regional pathology in SZ and ASD may be worth pursuing in future studies.

### Lateral and ventral temporal regions

Both SZ and ASD were associated with similar hypoactivation in temporal regions around the STS during ToM tasks; however ASD patients showed increased engagement in this region compared to controls and to SZ patients during FER.

Temporal lobe regions near the STS are thought to be involved in the representation of biologically salient cues relating to goal-directed movement [22] including the configural and changeable properties of human faces [56]. Processing of this information in regions around the STS is thought to assist in formulating attributions about the intentional states of others [23], [57].

Our results suggest that processing information relating to ToM tasks was associated with similar reduction in engagement within these regions both in SZ and ASD. However, we also found evidence that STS engagement may differ between disorders based on stimulus type, as ASD patients showed overactivation in these regions during FER. Reduced activation in regions around the STS (extending to the fusiform gyrus) has been noted in ASD patients during person identification tasks (i.e. memorising and recalling information about the identity of a face); however person identification is not required in facial emotion recognition paradigms which rely on decoding the “social” (i.e. emotional) cues conveyed by facial expression. Our results resonate with previous reports that patients with ASD rely excessively on facial feature extraction during facial processing [51] and suggest that they probably use a “logical” rather than a “social” approach to dealing with human facial expressions. Our findings reinforce the dependence of functional changes in ASD on the type of stimulus and also the specific task requirements which would be useful in informing and refining future study designs.

### Thalamus

Decreased engagement of the thalamus during social cognition was found mostly in connection to SZ, implicating the dorsomedial and pulvinar nuclei. The thalamus provides a nodal link for multiple functional circuits coordinating the flow of information in a range of higher cognitive and sensory processes. The pulvinar is is considered a “higher order” nucleus because of its widespread bidirectional cortical connections; it is directly involved in visual perception, particularly in directing and maintaining attention towards salient stimuli. The involvement of the dorsomedial thalamus in social cognition is mostly considered within the context of its general contribution to executive functions through its connections with the prefrontal cortex. Extensive thalamic pathology both in terms of microstructure, gross anatomy and functional engagement have been consistently reported in SZ [49]. Our findings extend this literature to include thalamic dysfunction to social cognition tasks.

### Methodological considerations

Although Activation Likelihood Estimation represents a powerful approach for the meta-analytic treatment of neuroimaging data, a number of factors should be considered in the interpretation of the current set of findings. First, comparison of neuroimaging studies between SZ and ASD is complicated by the variability of the activation paradigms used. We attempted to minimise this by grouping together paradigms that map on to different domains of social cognition and then examining them separately. This was possible for studies investigating attribution of affective states using facial expressions. However, given the variability of the ToM paradigms we followed the approach of other meta-analytic studies, which have also pooled several related domains of cognition together [31], [58]. Second, we accepted the results of individual original studies as reported, since ALE analyses do not allow for weighting based on the threshold of significance employed in each original study. Some studies have reported coordinates extracted from pre-specified regions of interest; this may have inflated the weight assigned to the findings regarding the amygdala and fusiform gyrus (FER studies), and medial prefrontal cortex, cingulate and STS (ToM studies). Third, ASD and SZ patients differ in their symptom profiles, with psychotic symptoms being predominantly associated with the diagnosis of SZ. Previous studies have suggested that the presence or absence of positive symptoms may contribute to the distribution and degree of functional disruption in SZ during social cognition [59]. The contribution of psychotic symptoms to the present findings is unclear. Nevertheless, the majority of SZ studies included patients that would be generally regarded as being in remission. Fourth, we have documented effects of antipsychotic medication on signal differences in several brain regions; however, these effects are predominantly ameliorative (i.e. reduced the effect size of group differences), and are thus unlikely to account for the differences observed between groups. Fifth, although gender differences have been found during social cognition tasks [58] it was not possible to examine this directly because the predominance of male participants in ASD studies did not allow gender-specific analysis of the available data. Fifth, age differences between the diagnostic groups may have influenced our findings, particularly in ToM tasks, where ASD patients were significantly younger than SZ patients. Indeed, an effect of age was observed in prefrontal regions, favouring SZ. Sixth, the average sample size per study was generally small. This suggests that individual studies may be prone to Type II errors, but also that our results represent the most robustly replicated group differences. Finally, our meta-analysis provides an estimate of how consistently clusters of differential activation occur in particular brain regions when comparing groups of individuals (e.g. SZ vs. controls), and not the mean activation difference in these regions. Therefore traditional measures of heterogeneity and publication bias that are based on the effect size of group differences are not applicable.

### Conclusions

In sum, the present results shed light on the neural correlates of social cognition in SZ and ASD and identify differences and similarities between the disorders. In the context of the current debate with regard to diagnostic boundaries, we hope that these findings will facilitate the formulation of new pathophysiological hypotheses and aid the design of future studies.

## Supporting Information

Figure S1 **PRISMA Flow Diagram.**
(DOC)

Table S1 **PRISMA Checklist.**
(DOC)
