# Supplementary material for: Autism Spectrum Disorders and Schizophrenia: Meta-Analysis of the Neural Correlates of Social Cognition
Source: PLoS One. 2011 Oct 5;6(10):e25322. doi: 10.1371/journal.pone.0025322 (PMC3187762; doi:10.1371/journal.pone.0025322)
Supplement: Figure S1 — PRISMA Flow Diagram. (DOC) [file pone.0025322.s001.doc]

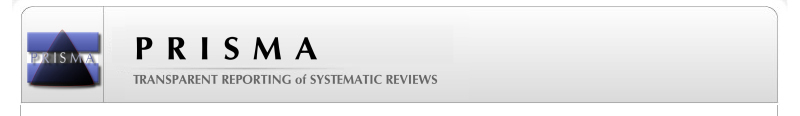
PRISMA 2009 Flow Diagram

**Screening**

**Included**

**Eligibility**

**Identification**

Records identified through database searching
(n = 415 )

Additional records identified through other sources
(n = 0 )

Records after duplicates removed
(n = 413 )

Records screened
(n = 413 )

Records excluded
(n = 337 )

Full-text articles assessed for eligibility
(n = 76 )

Full-text articles excluded, with reasons*
(n = 43 )

Studies included in qualitative synthesis
(n = 33 )

Studies included in quantitative synthesis (meta-analysis)
(n = 33 )

*Reasons:

- Lack of report of relevant between group contrasts or coordinates (n=24)

- Assessment of neuropsychological domains other than social cognition (n=12)

- FER contrasts not covering negatively valenced conditions (n=1)

- FER studies not using neutral faces as baseline (n=5)

- Imaging technique other than fMRI (n=1)
